# Association Between Metabolic Syndrome, Its Components, and Knee Osteoarthritis in Premenopausal and Menopausal Women: A Pilot Study

**DOI:** 10.7759/cureus.26726

**Published:** 2022-07-10

**Authors:** Marko Nemet, Tatjana Blazin, Stefan Milutinovic, Tatjana Cebovic, Dragana Stanojevic, Jelena Zvekic Svorcan

**Affiliations:** 1 Internal Medicine, University of Novi Sad, Faculty of Medicine, Novi Sad, SRB; 2 Internal Medicine, Beaumont Hospital, Royal Oak, USA; 3 Internal Medicine, Florida State University College of Medicine, Fort Myers, USA; 4 Department of Biochemistry, University of Novi Sad, Faculty of Medicine, Clinical Center of Vojvodina, Novi Sad, SRB; 5 Clinic for Cardiology, Clinical Center Nis, Novi Sad, SRB; 6 Department of Medical Rehabilitation, University of Novi Sad, Faculty of Medicine, Special Hospital for Rheumatic Diseases, Novi Sad, SRB

**Keywords:** hyperglycaemia, hypercholesterolemia, hypertriglyceridemia, obesity, osteoarthritis, metabolic syndrome

## Abstract

Objective: This pilot study aimed to determine the correlation of metabolic syndrome (MetS), its components, and increased LDL (low-density lipoprotein) and total cholesterol levels with osteoarthritis (OA). In addition, our goal was to establish the association between MetS and the degree of handicap measured by the Lequesne index of functionality and severity of knee osteoarthritis.

Materials and methods: The pilot study included 25 subjects with knee OA and 19 subjects without knee OA. All subjects were menopausal or premenopausal women. MetS was diagnosed according to the National Cholesterol Education Program, Adult Treatment Panel III. OA was diagnosed if Kellgren-Lawrence ≥ 2.

Results: MetS was detected in 80% of subjects with OA. In the non-OA group, MetS was detected in 26% of subjects. The difference in MetS prevalence between the two groups was significant (p=0.000). The presence of each MetS component was significant in the OA group, except for central obesity, which presence was marginally significant (p=0.054). Prevalence of increased total (p=0.019) and LDL cholesterol (p=0.000) was also significant in the OA group. A significant difference between OA and the non-OA group was detected in the prevalence of all five MetS components (p=0.016). In the OA group, the Lequesne index of functionality and severity of knee osteoarthritis was not significantly altered between subjects with and without MetS.

Conclusion: Metabolic syndrome, its components, increased LDL, and total cholesterol are correlated with osteoarthritis in premenopausal and menopausal women. MetS is not correlated with the degree of handicap in the knee joint measured by the Lequesne index.

## Introduction

Metabolic syndrome (MetS) is a set of interrelated biochemical, physiological and metabolic disorders that are primarily associated with an increased risk of coronary artery disease and type 2 diabetes mellitus [1]. These risk factors include abnormal laboratory findings of dyslipidemia and glucose intolerance, arterial hypertension, and central obesity. According to the National Cholesterol Education Program, Adult Treatment Panel III (NCEP ATP III) definition, MetS is diagnosed if three of the following five conditions are present: central obesity, hypertriglyceridemia, low high-density lipoprotein (HDL) cholesterol, fasting hyperglycemia, and arterial hypertension [2].

As recently reported by the National Health and Nutrition Examination Survey, MetS affects approximately a fifth of the US adult population [3]. The high incidence of this disorder led researchers to determine the association and impact of MetS on diseases and conditions other than coronary artery disease and type 2 diabetes mellitus.

Among rheumatic diseases, osteoarthritis (OA) is the most common and is the leading cause of disability in people over the age of 40. It is a degenerative disease of the synovial joints characterized by hyaline cartilage degradation, synovitis, and subchondral bone remodeling [4]. Regarding its high incidence, many researchers investigated possible associations between OA and MetS. OA is a degenerative disease of the synovial joints characterized by hyaline cartilage degradation, synovitis, and subchondral bone remodeling [4].

Obesity was described to be the primary cause of OA due to increased load on the joints since mechanical damage and wear and tear of the joints are considered to cause OA. However, since hand OA occurs five to eight times more often in obese individuals, we can conclude that the load due to increased body weight in obese people is not the only factor that contributes to OA development [5]. This finding led to the definition and identification of other risk factors and causes of OA, including a metabolic phenotype of OA, also known as MetS-linked OA. 

Zhuo et al. established OA as the fifth component of MetS after recognizing metabolic dysfunction in the pathophysiology of OA and its association with MetS and its components [6]. Several theories and hypotheses described how components of MetS can affect the pathogenesis and course of OA. Arterial hypertension leads to subchondral ischemia, while dyslipidemia leads to ectopic lipid deposition inside the chondrocytes. Next, hyperglycemia results in oxidative stress and low-grade inflammation, which ends in cartilage destruction. Also, central obesity can contribute to OA pathogenesis via its endocrine function. Finally, increased levels of leptin and adiponectin in synovial fluid are associated with greater joint destruction [4,6].

The primary goal of this pilot study was to detect if there is any significant association between metabolic syndrome and its components with knee osteoarthritis in premenopausal and menopausal women. Additionally, the goal was to test the used research model in order to make further research more feasible and effective. As knee OA is more frequent and more severe in women [7], and as we lacked male patients, we limited our subjects to women only. In addition, our goal was to examine the association between elevated total and LDL cholesterol with knee osteoarthritis and to detect if there is any association between metabolic syndrome and the degree of handicap according to the Lequesne index.

## Materials and methods

The research was conducted at the Special Hospital for Rheumatic Diseases, Novi Sad, Serbia. Institutional ethics committee approval was obtained for the pilot study, approval number 14/22-4/1-19. The participants were enrolled in the pilot study between November 2019 and February 2020, following their admission to inpatient hospital care. The patients provided informed written consent to participate in this pilot study.

Study population

The pilot study included 44 premenopausal or menopausal women, aged from 45 to 75 years. After a thorough clinical and radiological assessment, subjects were divided into two groups, experimental and control. The experimental group comprised 25 subjects diagnosed with OA of one or both knee joints. The control group comprised 19 subjects without OA of the knee, matched to subjects with OA by age and gender. Patients with secondary knee OA caused by trauma, patients with a traumatic knee injury, patients with rheumatoid arthritis of the knee, and patients with other autoimmune disorders were excluded. No woman was or had previously been on a hormone replacement therapy (HRT). All the subjects completed a brief demographic questionnaire. Participants with OA completed the Lequesne index of functionality and severity of knee osteoarthritis. Height, weight, waist circumference, and blood pressure measurements were taken. In addition, a blood sample was collected from each of the subjects.

Laboratory methods

Laboratory tests were performed in the biochemistry laboratory of the Special Hospital for Rheumatic Diseases, Novi Sad. Following eight to 12 hours of night fasting, blood samples were collected from the subjects. Sample analysis was performed with a CS-T240 automatic analyzer (Dirui, Changchun, China; production date November 2017). All laboratory parameters were determined using kits provided by the manufacturer Dirui. 

Anthropometric measurements

Anthropometric measurements were performed in the morning. Height, weight, and waist circumference were taken. The waist circumference was measured using a centimeter-tape resistant to stretching, at half the distance between the highest point of the iliac crest and the lower edge of the lowest rib. The measurement was performed in a standing position, at the end of an unforced expiration. Body mass index (BMI) was calculated using the obtained values of measured anthropometric parameters.

Blood pressure

Blood pressure values were measured using a sphygmomanometer. The first measurement was performed after a five-minute rest in a sitting position and the second five minutes after the first. The mean value of the two measurements was calculated.

Metabolic syndrome

Metabolic syndrome is diagnosed according to the modified National Cholesterol Education Program, Adult Treatment Panel III (NCEP-ATP III) criteria, which determines at least three of the five factors (or medication use) [2]: central obesity, hypertriglyceridemia, decreased HDL-C, elevated fasting blood glucose, and hypertension (Table 1).

**Table 1:** Modified NCEP ATP III diagnostic criteria for metabolic syndrome in women NCEP ATP III: National Cholesterol Education Program, Adult Treatment Panel III; HDL: high-density lipoprotein From the Modified NCEP ATP III diagnostic criteria for metabolic syndrome [2].

Disorder	Criteria
Central obesity	Waist circumference for women ≥ 88 cm
Low HDL cholesterol	HDL-C < 1.3 mmol/L or specific pharmacological therapy
Hypertension	Systolic blood pressure ≥ 130 mmHg, or Diastolic blood pressure ≥ 85 mmHg, or specific pharmacological therapy
Hypertriglyceridemia	≥ 1.7 mmol/L, or specific pharmacological therapy
Elevated fasting blood glucose	≥ 6.1 mmol/L, or specific pharmacological therapy

Other lipid disorders

In addition to low HDL cholesterol and hypertriglyceridemia, which are components of MetS, elevated LDL cholesterol and elevated total cholesterol were also monitored. These disorders were diagnosed according to the reference values issued by the laboratory for the biochemistry of the Special Hospital for Rheumatic Diseases, Novi Sad, or in the case of lipid-lowering therapy use. Upper normal limits values of LDL and total cholesterol were 4.78 mmol/L and 5.70 mmol/L, respectively. 

Knee osteoarthritis

The Kellgren-Lawrence (K/L) scale was used to grade radiographic anteroposterior images of the knee joint [8]. If the grade was two or higher, OA was diagnosed. Subjects with OA completed the Lequesne index for knee osteoarthritis [9]. The index contains three segments: pain and discomfort, maximum walking length, and daily life difficulties. The patients answered questions from these three segments, and for each answer, points were given. The minimum sum of points per segment is zero, and the maximum is eight. The minimum total sum of points is zero, and the maximum is 24. The handicap can range from no handicap to extremely severe handicap, determined by the total points [9] (Table 2).

**Table 2:** Lequesne index of functionality and severity of knee osteoarthritis From the Lequesne index of functionality and severity of knee osteoarthritis [9].

Handicap	Total points
No	0
Mild	1 - 4
Moderate	5 - 7
Severe	8 - 10
Very severe	11 - 13
Extremely severe	≥ 14

Statistical analysis

Collected data were analyzed using the SPSS 20.0 software (IBM Corp., Armonk, NY, USA). Numerical features of observation are processed by standard procedures of descriptive and comparative statistics. Although most of the measures used met the criterion of a continuous (numerical) variable, the limited sample size allowed for the use of non-parametric methods. The Mann-Whitney U-test was used to determine the difference between the groups with and without OA. The difference in frequency (distribution) of the observed parameters was estimated using a non-parametric Chi-square test. The threshold for significance was set at 5% (p < 0.05).

## Results

Subjects' general characteristics

Table 3 presents the general demographic and clinical characteristics of the subjects in the experimental and control groups.

**Table 3:** Participants’ demographic and clinical characteristics * Man-Whitney U test; M, mean; SD, standard deviation; BMI, Body Mass Index; SBP, systolic blood pressure; DBP, diastolic blood pressure; LDL-C, LDL cholesterol; HDL-C, HDL cholesterol.

Parametar	Experimental group (n=25)	Control group (n=19)	U-test*	p-level
	M (SD)	M (SD)		
Age	65.20 (6.99)	62.47 (8.34)	191.5	.275
Height (m)	1.60 (0.05)	1.62 (0.06)	179.5	.167
Weight (kg)	80.73 (15.36)	70.92 (11.52)	145.0	.028
BMI (kg/m2)	31.48 (5.49)	26.96 (4.41)	123.5	.007
Waist circumference (cm)	103.44 (12.48)	90.37 (12.17)	108.0	.002
SBP (mmHg)	130.20 (19.12)	122.63 (20.77)	170.0	.105
DBP (mmHg)	78.40 (7.87)	75.00 (8.50)	191.0	.240
Fasting blood glucose(mmol/L)	6.58 (1.17)	5.45 (0.94)	92.5	.001
Total cholesterol (mmol/L)	6.42 (1.23)	6.09 (0.99)	209.0	.499
LDL-C (mmol/L)	3.79 (1.18)	3.61 (0.99)	215.0	.594
HDL-C (mmol/L)	1.80 (0.30)	1.84 (0.43)	221.0	.696
Triglycerides (mmol/L)	1.81 (0.53)	1.44 (0.58)	135.0	.015

Differences between the experimental and control groups were examined using the Mann-Whitney U test. The Mann-Whitney U test showed significant differences in values between the experimental and control groups in body weight (p = 0.028), BMI (p = 0.007), waist circumference (p = 0.002), fasting blood glucose (p = 0.001), and triglyceridemia (p = 0.015). Subjects in the experimental group had a significantly higher average value on each of the mentioned parameters than subjects in the control group (Table 3). In terms of age, height, systolic and diastolic blood pressure, total cholesterol, LDL, and HDL cholesterol, no significant difference between the subjects of the experimental and control groups were found.

The frequency of medication use for hypertension, hyperglycemia, and hyperlipidemia in the experimental and control groups is shown in Table 4.

**Table 4:** Pharmacological therapy of the experimental and the control group * Chi-square test

Parameter	Group		χ2*	p-level
	Experimental	Control		
	number (%)	number (%)		
Antihypertensive therapy			5.740	.017
no	8 (32.0%)	13 (68.4%)		
yes	17 (68.0%)	6 (31.6%)		
Therapy for hyperglycemia			4.287	.038
no	20 (80.0%)	19 (100.0%)		
yes	5 (20.0%)	0 (0.0%)		
Therapy for hyperlipidemia			7.362	.007
no	11 (44.0%)	16 (84,2%)		
yes	14 (56.0%)	3 (15.8%)		

The Chi-square test showed a significant difference in medication use between the experimental and control groups (antihypertensive therapy p = 0.017; therapy for hyperglycemia p = 0.038 and therapy for hyperlipidemia p = 0.007). Table 4 shows that antihypertensive therapy is used by a higher percentage of subjects in the experimental group (68%) than in the control group (32%). No one in the control group takes therapy for hyperglycemia, however, 20% of the experimental group subjects do. Additionally, a significantly higher percentage of subjects in the experimental group, 56%, take therapy for hyperlipidemia, compared to about 16% of subjects in the control group.

Association of metabolic syndrome and its components with knee osteoarthritis

Table 5 shows the presence of metabolic syndrome and its components in the experimental and control groups.

**Table 5:** Metabolic syndrome and its components in the experimental and the control group * Chi-square test; HDL: high-density lipoprotein

Parameter	Group		χ2*	p-level
	Experimental	Control		
	number (%)	number (%)		
Metabolic syndrome			12.681	.000
no	5 (20.0%)	14 (73.7%)		
yes	20 (80.0%)	5 (26.3%)		
Hypertension			7.150	.007
no	2 (8.0%)	8 (42.1%)		
yes	23 (92.0%)	11 (57.9%)		
Hyperglycemia			8.729	.003
no	10 (40.0%)	16 (84.2%)		
yes	15 (60.0%)	3 (15.8%)		
Hypertriglyceridemia			5.439	.020
no	7 (28.0%)	12 (63.2%)		
yes	18 (72.0%)	7 (36.8%)		
Central obesity			3.709	.054
no	4 (16.0%)	8 (42.1%)		
yes	21 (84.0%)	11 (57.9%)		
Low HDL cholesterol			3.877	.049
no	11(44.0%)	14 (73.7%)		
yes	14 (56.0%)	5 (26.3%)		

The Chi-square test demonstrated significant differences between the experimental and control groups in terms of metabolic syndrome frequency (p = 0.000) and the presence of each individual component of metabolic syndrome: arterial hypertension (p = 0.007), hyperglycemia (p = 0.003), hypertriglyceridemia (p = 0.020), central obesity (p = 0.054) and low HDL cholesterol (p = 0.049). A value of p = 0.054 in central obesity is interpreted as marginally significant. Also, it is observed that the subjects from the experimental group have a higher percentage of metabolic syndrome compared to the control group (80% vs. about 26%). Each of the individual components of the metabolic syndrome is present in a higher percentage in the experimental group than in the control group: arterial hypertension (92% vs. about 58%), hyperglycemia (60% vs. about 16%), hypertriglyceridemia (72% vs. about 37%), central obesity (84% vs. about 58%) and low HDL cholesterol (56% vs. about 26%).

Association of elevated total cholesterol and LDL cholesterol with knee osteoarthritis

Table 6 shows elevated total cholesterol and elevated LDL cholesterol in the experimental and control groups.

**Table 6:** Elevated total cholesterol and LDL cholesterol in the experimental and the control group * Chi-square test; LDL: low-density lipoprotein

Parameter	Group		χ2*	p-level
	Experimental	Control		
	number (%)	number (%)		
Elevated total cholesterol			5.519	.019
no	2 (8.0%)	7 (36.8%)		
yes	23 (92.0%)	12 (63.2%)		
Elevated LDL cholesterol			13.672	.000
no	7 (28.0%)	16 (84.2%)		
yes	18 (72.0%)	3 (15.8%)		

Table 6 shows significant differences between the experimental and control groups in the frequency of high total cholesterol (p = 0.019) and high LDL cholesterol (p = 0.000), as shown by the Chi-square test. It has been reported that 92% of subjects in the experimental group have high total cholesterol, compared to around 63% in the control group. Furthermore, 72% of the experimental group's subjects have high LDL cholesterol, compared to about 16% of the control group's subjects.

Association of metabolic syndrome and Lequesne index of functionality and severity of knee osteoarthritis

Table 7 shows the experimental group with osteoarthritis, divided into the subgroup without metabolic syndrome (five subjects, 20%) and the subgroup with metabolic syndrome (20 subjects, 80%). The results of the Lequesne index, which is used to assess knee joint functionality and the severity of knee osteoarthritis, were compared.

**Table 7:** Lequesne index of functionality and severity of knee osteoarthritis in the experimental group * Chi-square test; ** Mann-Whitney U test; M, mean; SD, standard deviation.

Lequesne index- categories	Metabolic syndrome		χ2*		p-level
	No	Yes			
	number (%)	number (%)			
Mild handicap (1-4)	1 (20.0%)	0 (0.0%)	5.577	.233	
Moderate handicap (5-7)	0 (0.0%)	3 (15.0%)			
Severe handicap (8-10)	0 (0.0%)	3 (15.0%)			
Very severe handicap (11-13)	1 (20.0%)	4 (20.0%)			
Extremely severe handicap (≥ 14)	3 (60.0%)	10 (50.0%)			
	M (SD)	M (SD)	U-test**		p-level
Lequesne index- numerical value	14.40 (7.07)	12.60 (3.98)	39.5		.475

## Discussion

In our pilot study, the presence of MetS was significantly higher among subjects with knee OA compared with subjects without OA. Additionally, individual components of metabolic syndrome: arterial hypertension, elevated fasting blood glucose (FBG), elevated triglycerides (TAG) levels, and low HDL cholesterol levels were also significantly more present in subjects with knee OA. The relationship between central obesity and knee OA was only marginally significant.

Previous studies that investigated the same subject have found that MetS was often associated with OA. This was confirmed by a recent meta-analysis that showed a significant bidirectional relationship between OA and MetS in cross-sectional studies. Also, the same study showed a higher incidence of OA in subjects with baseline MetS in prospective cohorts [10]. Conversely, current literature shows conflicting results regarding each component of MetS.

The Fasa Osteoarthritis Study [11] and a large-scale cross-sectional study from China [12] have shown a significant correlation between MetS and OA. Also, almost all MetS components were associated individually with OA in the aforementioned studies. In the former study [11], however, arterial hypertension failed to show any association with the risk of developing OA. On the other hand, in the latter study [12], the association between elevated FBG and OA became insignificant after adjustment for age, sex, and other possible confounding factors. In contrast to these, Yoshimura et al. [13] have found that after adjusting for potential confounding factors, arterial hypertension and impaired glucose tolerance were associated with a higher occurrence of OA. Accordingly, larger population-based studies would be helpful to determine these possible associations.

In a cross-sectional study performed by Lee et al. [14], a significant relationship was determined between MetS, arterial hypertension, central obesity, and OA. However, MetS was only associated with OA in women and not in men. A meta-analysis [15] from 2020 confirmed these findings by showing that there is a significant association between MetS and incidence of knee OA only in females. These results could be explained by a higher sensitivity to metabolic derangements in females when it comes to OA development [7].

The study from the US by Puenpatom et al. [16] reported that the association between MetS and OA was stronger in younger subjects and the link became weaker in older patients. This finding could point to the importance of MetS in the pathogenesis of OA in younger patients, where the classic wear-and-tear mechanism is less likely.

Several studies have shown that MetS and its components were associated with knee OA only before adjusting for BMI or weight [17,18]. A Korean cohort study [19] reported that fat mass was associated with cartilaginous injury of the knee joint. Likewise, this link became weak after adjusting for BMI. Results from these studies suggest that increased BMI, which corresponds to increased mechanical load on the knee joint is, in fact, an important factor that contributes to the development and progression of knee OA. Regarding the relatively small number of subjects in our pilot study, we were not able to assess the association between metabolic factors and OA after adjusting for BMI. Nevertheless, it could be assumed that the pathogenesis of OA of different joints is probably not the same, nor does it have the same risk factors. A Norwegian cohort study has shown that obesity was associated with hand and knee OA however, not with hip OA [20]. Similarly, a study by Sanchez-Santos et al. described a relationship of MetS with hand OA even after adjusting for BMI [18].

Our pilot study reported significant relationships between elevated total cholesterol (TC), LDL cholesterol levels, and knee OA. As TC and LDL-C are not included in MetS, there are fewer studies that investigated this topic. Mishra et al. [21] found that both elevated TC and elevated LDL-C are associated with OA. Similarly, a British cross-sectional study [22] and a German case-control study [23] reported that elevated TC was associated with knee OA. On the other hand, other cohort studies discarded this hypothesis [24,25]. However, increased gene expression of the receptor for ox-LDL particles in chondrocytes of the cartilage affected by OA was documented [6]. According to these results, we would suggest additional studies to establish the link between increased TC and LDL-C with the incidence, prevalence, and pathogenesis of OA. 

Other than MetS and its components, a cumulative effect of MetS components on OA was frequently described. Lee et al. [14] have shown that the risk for OA increases with a higher number of components of MetS present. The highest risk had women with four or five MetS components. Similar findings were observed in other studies [11,12,18]. In the Fasa Osteoarthritis Study [11] subjects with four or five components had a 20 and 31 times higher risk of acquiring OA, respectively, compared to the subjects with no MetS components. We were unable to investigate this phenomenon since our experimental and control groups had relatively fewer subjects.

Finally, we investigated the knee joint function in subjects with OA with MetS and in those without MetS. In order to determine the functionality of the knee joint, we utilized the Lequesne index. The average Lequesne index score was no different between the two groups. Also, we found no difference between the two groups when the subjects are allocated to categories according to the level of handicap. In contrast to our results, other studies [26,27] have found a positive correlation between MetS and Lequesne index score. Hewala et al. [26] described that MetS was significantly more present in subjects with very severe and extremely severe handicaps according to the Lequesne index score. These findings could be explained by the increased pain in the metabolic phenotype of OA since the pain limits the function of the joint. 

Since MetS is a well-determined risk factor for cardiovascular diseases, its association with OA should raise the index of clinical suspicion for possible cardiovascular comorbidities when diagnosing OA and vice versa. This is particularly important when it comes to the management of OA, where NSAIDs are frequently used. Thus, NSAIDs could raise the risk for cardiovascular disease (CVD) even higher, especially if there is a concomitant MetS or its components [28]. In addition, OA can significantly restrict physical activity and thus decrease the quality of life, especially in patients with cardiovascular diseases [29]. Moreover, the lack of physical activity in patients with OA could lead to or exacerbate individual MetS components and even result in MetS [30]. 

This pilot study has several limitations and the results must be interpreted with caution. Firstly, the study sample size is limited and there is an increased likelihood of type II error which decreases the power of the study. Secondly, as we conducted research only on women, the sample is not representative of the population with OA. 

## Conclusions

In conclusion, this pilot study found that MetS and its components are associated with knee OA in premenopausal and menopausal women. We also determined that there is an association between elevated TC and LDL-C with knee OA in our subjects. Finally, MetS was not associated with the score of the Lequesne index of severity for osteoarthritis.
